# Selection of Luteolin as a potential antagonist from molecular docking analysis of EGFR mutant

**DOI:** 10.6026/97320630014241

**Published:** 2018-05-31

**Authors:** George Oche Ambrose, Olanrewaju John Afees, Nwufoh Chika Nwamaka, Nzikahyel Simon, Adebo Adeola Oluwaseun, Tosin Soyinka, Alakanse Suleiman Oluwaseun, Seyi Bankole

**Affiliations:** 1 Centre for Bio computing and Drug Development, Adekunle Ajasin University, Ondo State; 2 Anatomy Department, University of Ilorin, Ilorin, Kwara State; 3 Faculty of Pharmaceutical Sciences Nnamdi Azikiwe University Agulu, Anambra State; 4 Chemistry Department, University of Uyo, Uyo, Akwa-Ibom State; 5 Chemical Pathology, Olabisi Onabanjo Teaching Hospital, Ogun State; 6 Biochemistry Department, University of Ilorin, Ilorin, Kwara State; 7 Babcock University Teaching Hospital, Ilesan, Ogun State

**Keywords:** EGFR mutant inhibitors, luteolin, docking

## Abstract

The life-threatening sides effect of the current EGFR mutant inhibitors (drugs) such as the eruption of rash which can be seen on the face, chest, back and even the trunk, diarrhea, nausea, vomiting, anorexia and stomatitis, necessitates the discovery of new potent and safe compounds as a chemo-therapeutic measure against lung cancer. Approximately about 10% of patients with Non-small cell lung cancer (NSCLC) in the US and about 35% in East Asia have tumor associated EGFR. These mutations occur within EGFR exon 18-21, which encodes a portion of the EGFR kinase domain and enables researchers to identify compounds that only recognizes and binds to the cancer cells. Thus, mutations in EGFR play a role as both biomarkers and rational targets for targeted therapy. In view of this, we out-source for the best-in -class inhibitor for this druggable target via computational tools.

The purpose of this study was to analyze the inhibitory potential of luteolin by computational docking studies. For this, three (3) flavone chemical compounds (phytochemicals) retrieved from literatures were screened for their inhibitory effects on the epidermal growth factor receptor (EGFR). Luteolin was the lead compound with a binding energy of -7.7 kcal/mol. Computational docking analysis was performed using PyRx, AutoDock Vina option based on scoring functions and the target was validated so as to ensure that the right target and appropriate docking protocol was used for this analysis.

## Background

The major cause of cancer-related deaths worldwide is the nonsmall cell lung cancer (NSCLC) [1]. Recent studies have shown that the development of epidermal growth factor receptor (EGFR)-targeted tyrosine kinase inhibitors causes a significant advances in patients with tumors harboring EGFR mutations. Thus, about 50% of Asian patients with NSCLC have EGFR mutations [2]. EGFR has become an important therapeutic target for the treatment of lung cancer because more than 60% of non- small cell lung carcinomas (NSCLCs) express EGFR [3].

Epidermal growth factor receptor (EGFR) is a transmembrane protein with cytoplasmic kinase activity, which transduces important growth factor signaling from the extracellular environment to the cell [3]. It functions largely by its role in promoting cell proliferation and opposing apoptosis thereby verified as a proto-oncogene [4]. The RAS-RAF-MEK-ERK MAPK pathway may be the most important pathway in mediating the biological response of the EGFR. Proto-oncogenes RAS and RAF are found in this pathway. A major therapeutic target in lung cancer therapy is MEK. ERK MAPK (mitogen-activated protein kinase) interacts with over a hundred substrates to initiate a wide array of physiological and pathological responses, including growth, proliferation, differentiation, migration, and inhibition of apoptosis [5, 6].

EGFR mutations occur at mutational "hotspots" in the extracellular region, the kinase domain, and the C-terminal tail [7]. Such mutant EGFR is overexpressed in about 40-80% of NSCLC [8, 9]. Recently, molecular targeted therapies have been developed and have provided a remarkable relevance to NSCLC patients with specific genetic mutations. In particular, NSCLC with mutation in the epidermal growth factor receptor (EGFR) gene are sensitive to EGFR inhibition with specific tyrosine kinase inhibitors (TKIs). EGFR-TKIs are efficacious in patients with NSCLC harboring EGFR mutations as demonstrated in prospective clinical trials [7, 8, 9, 10, 11, 12, 13].

Inhibitors that target the kinase domain of EGFR have been developed and are pharmacologically active. Of utmost importance, such tyrosine kinase inhibitors (TKIs) are especially effective in patients whose tumors harbor activating mutations in the tyrosine kinase domain of the EGFR gene. More recent trials have suggested that for advanced NSCLC patients with EGFR mutant tumors, initial therapy with a TKI instead of chemotherapy may be the best choice of treatment [3]. However, currently available EGFR TKIs such as erlotinib are associated with the incidences of alopecia, nausea, vomiting, neurotoxic symptoms, and myelosuppression. Common side effects of EGFR TKIs are folliculitis, diarrhea, dry skin, and fatigue [14]. This constitute the aim of the present study which involves the identification of novel phytochemicals that offers a better inhibitory effect against mutant EGFR with no side effects to the patients.

Flavones are non-essential nutrients that provide additive nutraceutical value to our diet. Their health beneficial activities have been historically recognized across different cultures [15]. Flavonoids, including flavones, have received increasing attention due to their anti-inflammatory, anti-microbial and anticancer activities. Most of the anti-inflammatory and antimicrobial activities attributed to flavones appears to be particular on their ability to regulate the Toll receptor (TLR)/NF_B axis [16]. Previous studies have shown that of the three (3) flovones considered in this present study; apigenin reduced breast cancer cell migration, by inhibiting mitogen activated protein kinases (MAPK), including ERK and JNK [17] while luteolin prevents inflammation and neuronal damage by reducing Rho GTPases activity and thus decreasing leukocyte migration [18]. In view of the health related benefit of flavones, this study aims at revealing the multi-target drug ability of luteolin in an attempt to identify a potent inhibitor of mutant EGFR with little or no side effects. This is achieved by utilizing in-silico approach, which provided a highquality interaction between the ligand (luteolin) and the receptor (EGFR). Luteolin was then channelled to Lipinski rule of five on ADMET (Adsorption, Distribution, Metabolism, Excretion and Toxicity) properties and was found to fulfill the rule of five on ADMET properties.

## Methodology

### Ligand selection and preparation

The chemical structures of three (3) phytochemicals (apigenin, luteolin and tangeretin) were obtained from PubChem compound database (https://pubchem.ncbi.nlm.nih.gov). The MOL SDF format of these ligands were converted to PDBQT file using PyRx tool to generate atomic coordinates and energy was minimized by optimization using the optimization algorithm at force field set at mmff94 (required) on PyRx.

### Accession and preparation of the target protein

The protein, mutant epidermal growth factor receptor (EGFR) was prepared by retrieving the three-dimension crystal structure of EGFR kinase domain in complex with ligand, 1-{3[2-chloro-4- {5-[2-[2-hydroxyethoxylethyl-5H-pyrrolo[3,2-d]pyrimidine-4- yl}amino}phenoxy]phenyl}-3-cyclohexylurea, (PDB: 3W2S) from RCSB PDB (http://www.rcsb.org/pdb/home/home.do) [19]. Subsequently, The bound complex molecules with the proteins were removed. The non-essential water molecules and all heteroatoms were removed using Pymol tool and Discovery studio 2017R2 respectively. The crystallized ligand was extracted (not removed) from the active site so as to reveal the grid coordinate around the binding pocket when viewed on pymol and Discovery studio 2017R2 visualizer.

### Accession and Preparation of the Standard

The standard compound used in the present study is the cocrystalized ligand of the EGFR receptor (PDB: 3W2S]. The structure of the standard (1-{3[2-chloro-4-{5-[2-[2- hydroxyethoxylethyl-5H-pyrrolo[3,2-d]pyrimidine-4- yl}amino}phenoxy]phenyl}-3 cyclohexylurea) (PDB Ligaind ID:2WR) extracted from the receptor's active site was converted to PDBQT file using PyRx tool to generate atomic coordinates and energy was minimized by optimization using the optimization algorithm at force field set at mmff94 (required) on PyRx.

### Molecular docking using PyRx

**Figure 3:**
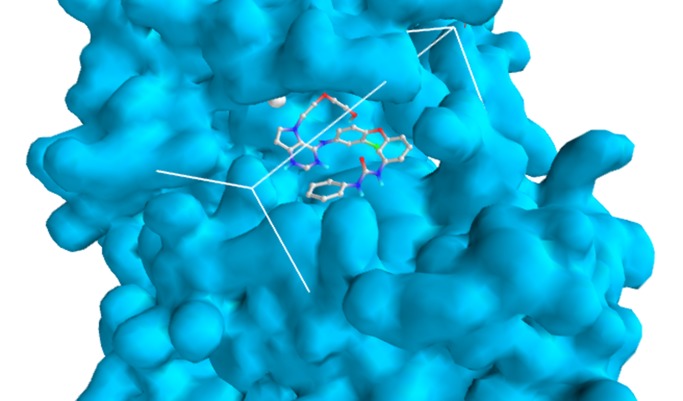
Grid box within which the ligand binds 4.359 x 8.0594 x 14.9283 along the X, Y, Z-axis.

Subsequent to receptor and ligands preparation, molecular docking analysis was performed using PyRx, AutoDock Vina option based on scoring functions. For our analysis we used the PyRx, AutoDock Vina exhaustive search docking function. After the minimisation process, the grid box resolution was centered at 4.359 x 8.0594 x 14.9283 along the x, y and z axes respectively at grid dimension of 25x 25 x 25 Å to define the binding site (Figure 3). The standard was first docked within the binding site of EGFR and the resulting interaction was compared with that of luteolin into the similar active sites using the same grid box dimension.

### Validation of docking results

The docking results obtained were validated with the blasting of the fasta sequence of the crystal structure of the human EGFR (ID: 3W2S), which was obtained from the protein data bank unto the online available ChEMBL Database (www.ebi.ac.uk/chembl/). The bioactivity generated by the database, having an inhibition of 4 and IC50 value of 115, was downloaded in txt format. The bioactivity was sorted out; missing or misplaced data were removed. Only 55 of the total 115 drug-like compounds were recovered. The compiled compounds were split and converted to 2D (in sdf format) by Data Warrior software (version 2) and converted to pdbqt format by PyRx tool. The ligands were docked into the binding domain of EGFR using PyRx AutoDock vina scoring function. A correlation coefficient graph was plotted between the docking scores of the 55 compounds generated and their corresponding PCHEMBL_VALUE (experimentally determined) values. Spearman Rank correlation co efficient graph was plotted on R language to obtain the correlation (R^2^) between the dockings results of the ChEMBl's compounds and their corresponding experimentally generated results.

## Results & Discussion

Epidermal growth factor receptor (EGFR) is a transmembrane protein with cytoplasmic kinase activity that transduces important growth factor signaling from the extracellular environment to the cell. It functions largely by its role in promoting cell proliferation and opposing apoptosis thereby verified as a proto-oncogene [3, 4]. It is therefore reasonable to think that inhibiting EGFR, represents a sound pharmacological approach.

In the present study, three (3) phytocompounds (flavones) present in plants and obtained from literatures were docked into the binding pocket of EGFR for their EGFR inhibitory (antagonistic) properties. Luteolin was discovered as the lead compound with the binding energy of -7.7 kcal/mol while that of apigenin and tangeretin are -7.1 and -6.1 kcal/mol respectively (Table 1). Subjecting it to the Lipinski's rule of five, afterwards the lead compound assessed the drug-likeness of luteolin, luteolin violated none of the rules, describes its bioavailability and binding potential (Table 4).

Luteolin, the lead compound has a binding energy of -7.7 kcal/mol, while the standard compound has binding energy of - 7.2 kcal/mol (Table 1). The highest binding energy (-7.7 kcal/mol) attributed to luteolin in this regard is believed to be as a result of its chemical interactions at the receptor's active site (Table 2; Figure 6), which includes: Ten (10) Hydrogen bonds involving K-875 and R-841 residues; Twelve (12) Hydrophobic interactions involving F-723 and A-722 residues; Twelve (12) Electrostatic interactions involving K-745 and ASP-855 residues.

While, that of the co-crystallized ligand (PDB Ligaind ID: 2WR) which serves as the standard presents with the following chemical interactions at the binding pocket (Table 3; Figure 6). Eight (8) Hydrogen bonds involving C-797 and (3) D-855 residues; Fourteen (14) Hydrophobic interactions involving K- 723, C-797, R-841 and A-722 residues; Two (2) Electrostatic interaction involving R-841 residue.

Hydrogen (H)-bonds potentiates diverse cellular functions by facilitating molecular interactions. In order words, hydrogen bonds are considered to be facilitators of protein-ligand binding [20, 21]. Previous studies have shown that synergistic receptorligand H-bond pairings potentiate high-affinity binding which corresponding to an increase in binding affinity [22]. It is obvious then that the higher binding affinity of luteolin to the binding pocket of mutant EGFR when compared to that of the cocrystallized ligand is attributed to the number of hydrogen bonds present in luteolin (10 hydrogen bonds) as compared to the standard (8 Hydrogen bonds).

**Figure 4:**
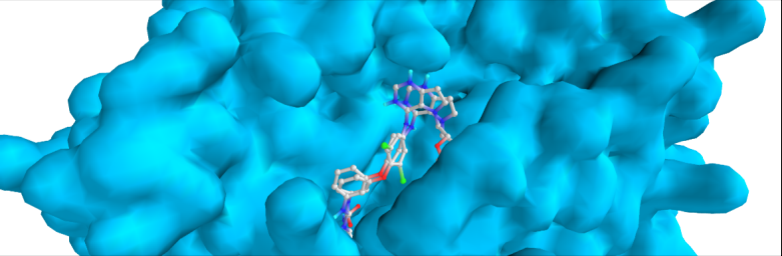
Validation of docking: Comparability of the re-docked binding mode and the co-crystallized pose of W2R with the accompany residues of mutant EGFR binding pocket using PyRx.

**Figure 6:**
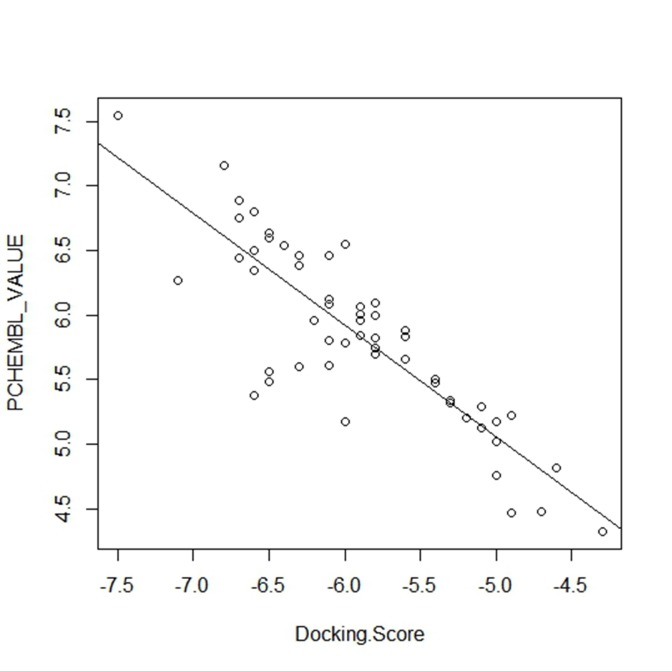
Correlation coefficient graph of docking scores of various antagonists of the EGFR and their corresponding experimental pIC50 (pchembl_values) values. The antagonists (compounds) and their corresponding pIC50 (experimentally derived IC50) were downloaded from the ChemBL database, the strong correlation (0.7319) between the docking scores and pIC50 shows that computer can reproduce experimental values and this gives credence to the docking scores generated, in the present study.

We validated the accuracy of our docking protocol by re-docking the co-crystallized ligand ((PDB Ligand ID: 2WR)) back into the binding pocket of the mutant EGFR (PDB: 3W2S). As stated, the re-docked pose overlapped almost totally with the experimental orientation, indicating that Autodock vina on PyRx re-docked the co crystallized ligand, with a very high accuracy, back into the binding pocket of the EGFR, this reveals that our docking methodology was reliable and the docking scores obtained are correct (Figure 4). The reliability of our docking scores was further validated using the online available ChEMBL Database, the Fasta sequence of the crystal structure of mutant EGFR (ID: 3W2S) was blasted on www.ebi.ac.uk/chembl/. The compounds obtained from the search were docked into the binding site of the mutant EGFR, a correlation coefficient graph plotted between the docking scores of the compounds generated and their corresponding ChEMBL's Pchem values (experimentally determined IC50). This showed a strong correlation coefficient between the docking scores and the experimentally derived data in the present study which gave credence to the fact that computational experiment can replicate experimental data at least in this present study and that our docking scores, using PyRx AutoDock Vina algorithm is dependable (Figure 6).

## Conclusion

Docking studies and ADMET evaluation of luteolin with EGFR showed that this ligand is a drug-gable molecule, which docks well with mutant EGFR tRet. Therefore, luteolin molecule plays an important role in inhibiting mutant EGFR and thus should be implicated as a potential agent in cancer therapy.

## Figures and Tables

**Table 1:** Energy and RMSD values obtained during docking analysis

S/N	Complex	Binding energy (From PyRx)	RMSD/UBa	RMSD/LBb
1	Luteolin	-7.7	0	0
2	Apigenin	-7.1	0	0
3	Tangeretin	-6.9	0	0
4	W2R	-7.2	0	0
RMSD/UB: Root mean square deviation/upper bond; RMSD/LB: Root mean square deviation/lower bond; EGFR: Epidermal growth factor receptor				

**Table 2:** Interaction table showing the various chemical interactions of luteolin within the binding pocket

Name	Category	Types
A:R 841:HH22-LUTEOLIN:O	Hydrogen Bond	Conventional Hydrogen Bond
Luteolin:H - A:K 875:O	Hydrogen Bond	Conventional Hydrogen Bond
A:K 745:NZ - LUTEOLIN	Electrostatic	Pi-Cation
A:D 855:OD2 - LUTEOLIN	Electrostatic	Pi-Anion
A:D 855:OD2 - LUTEOLIN	Electrostatic	Pi-Anion
A:F 723 - LUTEOLIN	Hydrophobic	Pi-Pi T-shaped
LUTEOLIN - A:A 722	Hydrophobic	Pi-Alkyl
A:K 745:HZ1 - A:D 855:OD1	Hydrogen Bond; Electrostatic	Salt Bridge
A:K 745:HZ2 - A:D 855:OD2	Hydrogen Bond; Electrostatic	Salt Bridge
A:R 841:HH22 - LUTEOLIN:O	Hydrogen Bond	Conventional Hydrogen Bond
LUTEOLIN:H - A:K 875:O	Hydrogen Bond	Conventional Hydrogen Bond
A:F 723 - LUTEOLIN	Hydrophobic	Pi-Pi T-shaped
LUTEOLIN - A:A 722	Hydrophobic	Pi-Alkyl
A:K 745:HZ1 - A:D 855:OD1	Hydrogen Bond; Electrostatic	Salt Bridge; Attractive ChRe
A:K 745:HZ2 - A:D 855:OD2	Hydrogen Bond; Electrostatic	Salt Bridge; Attractive ChRe
A:R 841:HH22 - LUTEOLIN:O	Hydrogen Bond	Conventional Hydrogen Bond
LUTEOLIN:H - A:K 875:O	Hydrogen Bond	Conventional Hydrogen Bond
A:K 745:NZ - LUTEOLIN	Electrostatic	Pi-Cation
A:D 855:OD2 - LUTEOLIN	Electrostatic	Pi-Anion
A:D 855:OD2 - LUTEOLIN	Electrostatic	Pi-Anion
A:F 723 - LUTEOLIN	Hydrophobic	Pi-Pi T-shaped
LUTEOLIN- A:A 722	Hydrophobic	Pi-Alkyl
A:K 745:HZ1 - A:D 855:OD1	Hydrogen Bond; Electrostatic	Salt Bridge; Attractive ChRe
A:K 745:HZ2 - A:D 855:OD2	Hydrogen Bond; Electrostatic	Salt Bridge; Attractive ChRe
A:R 841:HH22 - LUTEOLIN:O	Hydrogen Bond	Conventional Hydrogen Bond
LUTEOLIN:H - A:K 875:O	Hydrogen Bond	Conventional Hydrogen Bond
A:K745:NZ - LUTEOLIN	Electrostatic	Pi-Cation
A:D 855:OD2 - LUTEOLIN	Electrostatic	Pi-Anion
A:D 855:OD2 - LUTEOLIN	Electrostatic	Pi-Anion
A:F723 - LUTEOLIN	Hydrophobic	Pi-Pi T-shaped
LUTEOLIN - A:A 722	Hydrophobic	Pi-Alkyl
A:F 723 - LUTEOLIN	Hydrophobic	Pi-Pi T-shaped
LUTEOLIN - A:A722	Hydrophobic	Pi-Alkyl
A:K 745:HZ1 - A:D 855:OD1	Hydrogen Bond; Electrostatic	Salt Bridge; Attractive ChRe
A:K 745:HZ2 - A:D 855:OD2	Hydrogen Bond; Electrostatic	Salt Bridge; Attractive ChRe
A:R 841:HH22 - LUTEOLIN:O	Hydrogen Bond	Conventional Hydrogen Bond
LUTEOLIN:H - A:K 875:O	Hydrogen Bond	Conventional Hydrogen Bond
A:K 745:NZ - LUTEOLIN	Electrostatic	Pi-Cation
A:D 855:OD2 - LUTEOLIN	Electrostatic	Pi-Anion
A:D 855:OD2 - LUTEOLIN	Electrostatic	Pi-Anion
A:F 723 - LUTEOLIN	Hydrophobic	Pi-Pi T-shaped
LUTEOLIN - A:A 722	Hydrophobic	Pi-Alkyl
R: Arg, A:Ala, F:Phe, K:Lys, D:Asp		

**Table 3:** Interaction table showing the chemical interactions of the co-crystalized within the binding pocket

Name	Category	Types
A:C797:SG - N:W2R:N	Hydrogen Bond	Conventional Hydrogen Bond
N:W2R:H - A:D 855:OD2	Hydrogen Bond	Conventional Hydrogen Bond
A:R 841:NH2 - N:W2R	Electrostatic	Pi-Cation
A:F 723 - N:W2R:Cl	Hydrophobic	Pi-Alkyl
N:W2R - A:C 797	Hydrophobic	Pi-Alkyl
N:W2R - A:C 797	Hydrophobic	Pi-Alkyl
N:W2R - A:R 841	Hydrophobic	Pi-Alkyl
N:W2R - A:A 722	Hydrophobic	Pi-Alkyl
A:C 797:SG - N:W2R:N	Hydrogen Bond	Conventional Hydrogen Bond
N:W2R:H - A:D 855:OD2	Hydrogen Bond	Conventional Hydrogen Bond
A:C 797:SG - N:W2R:N	Hydrogen Bond	Conventional Hydrogen Bond
N:W2R:H - A:D 855:OD2	Hydrogen Bond	Conventional Hydrogen Bond
A:F 723 - N:W2R:Cl	Hydrophobic	Pi-Alkyl
N:W2R - A:C 797	Hydrophobic	Pi-Alkyl
N:W2R - A:C 797	Hydrophobic	Pi-Alkyl
N:W2R - A:R 841	Hydrophobic	Pi-Alkyl
N:W2R - A:A 722	Hydrophobic	Pi-Alkyl
A:C 797:SG - N:W2R:N	Hydrogen Bond	Conventional Hydrogen Bond
N:W2R1101:H - A:D 855:OD2	Hydrogen Bond	Conventional Hydrogen Bond
A:R 841:NH2 - N:W2R1101	Electrostatic	Pi-Cation
A:F 723 - N:W2R1101:Cl	Hydrophobic	Pi-Alkyl
N:W2R1101 - A:C 797	Hydrophobic	Pi-Alkyl
N:W2R1101 - A:C 797	Hydrophobic	Pi-Alkyl
N:W2R1101 - A:R 841	Hydrophobic	Pi-Alkyl
R: Arg, A:Ala, F:Phe, K:Lys, D:Asp, C:Cys		

**Table 4:** Lipinski's drug-like properties of luteolin: The rule describes drug candidate's pharmacokinetics in the human body which also including their absorption, distribution, metabolism, and excretion ("ADME") using an online server (http://www.scfbio-iitd.res.in/)

Molecular Properties	Lipinski's rule of Five	Luteolin drug-like properties
Molecular Mass	<500	286.239
Hydrogen bond Acceptor	<10	6
Hydrogen bond Donor	<5	4
LogP	<5	1.55856
Molar Refractivity	Between 40-130	62.666996
Topological Polar surface area	<140Å^2^	107

**Figure 1:**
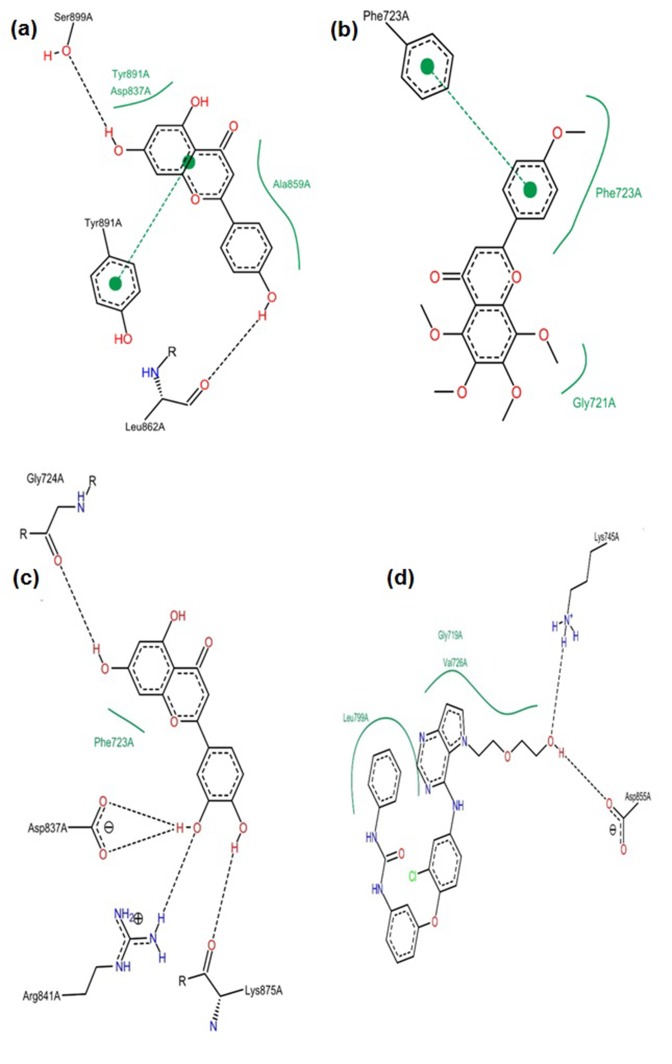
Pose view (a) Apigenin (b) Tangeretin (c) Luteolin (d) W2R (Co-crystallized ligand)

**Figure 2:**
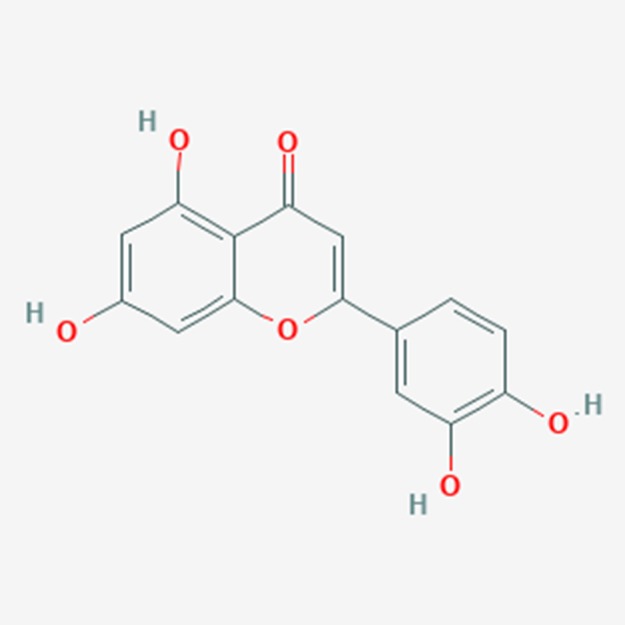
Structure of Luteolin

**Figure 5:**
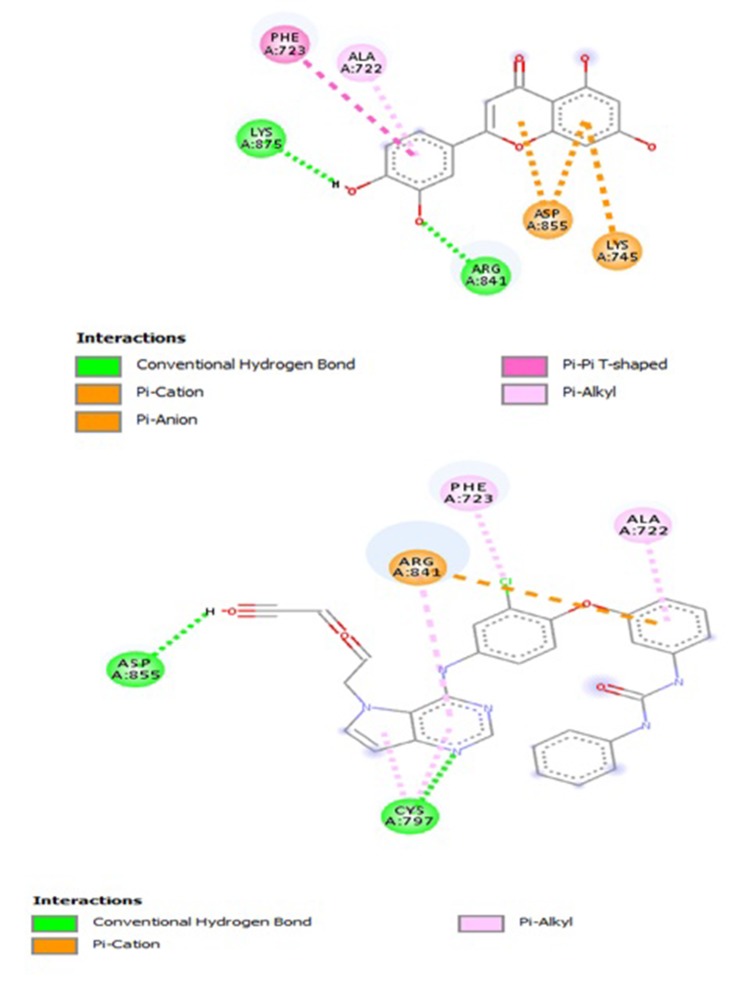
2D interactions of ligands within the binding pocket (a) luteolin (b) W2R
